# Genome-Wide Distribution, Expression and Function Analysis of the U-Box Gene Family in *Brassica oleracea* L.

**DOI:** 10.3390/genes10121000

**Published:** 2019-12-02

**Authors:** Dengke Hu, Qinqin Xie, Qianying Liu, Tonghong Zuo, Hecui Zhang, Yizhong Zhang, Xiaoping Lian, Liquan Zhu

**Affiliations:** College of Agronomy and Biotechnology, Southwest University, Chongqing 400715, China

**Keywords:** *Brassica oleracea* L., U-box, self-incompatibility, family expansion, evolution, RT-qPCR

## Abstract

The plant U-box (PUB) protein family plays an important role in plant growth and development. The U-box gene family has been well studied in *Arabidopsis thaliana*, *Brassica rapa*, rice, etc., but there have been no systematic studies in *Brassica oleracea*. In this study, we performed genome-wide identification and evolutionary analysis of the U-box protein family of *B. oleracea*. Firstly, based on the Brassica database (BRAD) and the Bolbase database, 99 *Brassica* *oleracea* PUB genes were identified and divided into seven groups (I–VII). The BoPUB genes are unevenly distributed on the nine chromosomes of *B. oleracea*, and there are tandem repeat genes, leading to family expansion from the *A. thaliana* genome to the *B. oleracea* genome. The protein interaction network, GO annotation, and KEGG pathway enrichment analysis indicated that the biological processes and specific functions of the BoPUB genes may mainly involve abiotic stress. RNA-seq transcriptome data of different pollination times revealed spatiotemporal expression specificity of the BoPUB genes. The differential expression profile was consistent with the results of RT-qPCR analysis. Additionally, a large number of pollen-specific cis-acting elements were found in promoters of differentially expressed genes (DEG), which verified that these significantly differentially expressed genes after self-pollination (SP) were likely to participate in the self-incompatibility (SI) process, including gene encoding ARC1, a well-known downstream protein of SI in *B. oleracea*. Our study provides valuable information indicating that the BoPUB genes participates not only in the abiotic stress response, but are also involved in pollination.

## 1. Introduction

Plant self-incompatibility exists mainly to prevent inbreeding and promote outcrossing [1], while the decline of self-inbreeding makes outcrossing more urgent. The signal transduction of sporphyric self-incompatibility responses in *Brassica oleracea* has been uncovered. When self-fertilized pollen falls on the stigma, the S-locus cysteine rich protein (SCR) in the pollen, combined with the extracellular domain of the S-locus receptor kinase (SRK) of the stigma mastoid cell, the SRK intracellular kinase domain, and the M-locus protein kinase (MLPK) are phosphorylated. At this time, arm repeat–containing protein 1 (ARC1) is located near the membrane of the nucleus [2]. The ARC1 arm repeat region interacts with the SRK kinase domain and MLPK to cause ARC1 activation, and its N-terminus then binds to Exo70A1. With the assistance of E1 and E2, Exo70A1 is ubiquitinated to a certain extent, guided by the U-box at the N-terminus of ARC1 to 26S proteasome, and degraded [3]. It then causes physiological processes, such as *MOD* (an aquaporin-like coding gene) protein closure, which ultimately inhibits the growth of the self-pollen tube and pollen germination, resulting in a self-incompatibility reaction [4]. In this process, the U-box domain is required for ARC1 localization to the 26S proteasome/CSN [5], and the proteins labeled by ARC1 ubiquitination can only be degraded by the action of 26S proteasome/CSN [4]. This further indicates the importance of the U-box gene family in the self-incompatibility signal transduction of *B. oleracea*.

The U-box is a highly conservative E2 interaction module and the definition domain of plants [6]. The U-box gene family has been identified in a variety of plants. Wiborg et al. identified 64 U-box proteins in *Arabidopsis thaliana* [7], Zeng et al. identified 77 U-box proteins in rice [8], and Hu et al. identified 91 U-box proteins in bananas [9]. Wang et al. identified 101 putative PUB genes in the *B. rapa* genome and compared them with 15 other representative plants [10]. A large number of experimental studies have also revealed the function of the U-box in various biological pathways. Several studies have shown that U-box protein (PUB) plays an important role in drought stress, ABA signaling, and vesicle and membrane protein transport regulation [6,11,12]. Both rice OsPUB15 and *A. thaliana* AtPUB17 were confirmed to be positive regulators of cruciferous cell death and defense [13,14]. Ubiquitin ligases AtPUB46 and AtPUB48 play key roles in drought and oxidative stress, and heat tolerance of *A. thaliana* [15,16], and the rice U-box E3 ubiquitin ligase OsPUB24 is crucial for fine-tuning of brassinosteroid (BR) response in rice [17]. Recently, it has been found that PUB is not only involved in the interaction of kinase domain [18], but also interacts with different types of heterotrimeric GTP-binding proteins (G-proteins). For example, the E3 ligases PUB4 and PUB2 interact with G-protein to play a role in cytokinin and development [19]. The above studies indicate that plant U-box E3 ubiquitin ligase (PUB) not only plays the role of self-incompatibility, but also plays an important role in immune response, adaptation to abiotic stress, and other stress responses, which have been extended to the regulation of the development process [6].

The U-box protein family has been well-analyzed in a variety of plants, but while many of the *B. oleracea* have been analyzed for individual genes to date, systematic studies based on the whole genome are rare. Conducting genome-wide evolution and distribution studies is an important means of understanding the complex functions of BoPUB. In this study, based on RNA-seq transcriptome and quantitative PCR, we performed a systematic bioinformatics analysis of the U-box gene family of *B. oleracea*, including phylogenetic evolution, conserved motif, gene structure, chromosome localization, tandem repeat events, protein interaction network, GO annotation, and KEGG enrichment analysis. In addition, we also classified the U-box gene family of *B. oleracea* and screened differentially expressed genes, on the basis of which we completed the cis-acting element analysis. This study provides important information for further revealing the evolutionary analysis and functional study of U-box genes in *B. oleracea*.

## 2. Materials and Methods

### 2.1. Plant Materials and Treatment

The plant materials used in this study included different haplotype advanced generation inbred lines A4 and F1, which were planted by the Cruciferae Vegetable Research Institute of the Southwest University College of Landscape and Horticulture. They were all grown under natural conditions. A4 is a SRK28 haplotype and F1 is a SRK7 haplotype. Both A4 and F1 are self-incompatible *B. oleracea* varieties. The pollen of the A4 material and the A4 stigma of the emasculation were self-pollinated, and is referred to as SP. The pollen of the F1 material and the A4 stigma of the emasculation were cross-pollinated, and is referred to as CP. After treatment at different pollination times, A4 pistil material was quickly removed and stored in liquid nitrogen at −80 °C for later use.

### 2.2. Retrieval and Identification of U-Box Family Genes in B. oleracea

In order to identify and classify the gene members of the U-box family of *B. oleracea*, 103 BoU-box gene family members were first searched for in the *Brassica* database (http://brassicadb.org/brad/) and the Bolbase database (http://ocri-genomics.org/bolbase/). Subsequently, after SMART (https://smart.embl.de/) online analysis, it was found that four genes clearly did not have a U-box structure. For the accuracy of the results, we downloaded the genome-wide sequence from the Bolbase database and downloaded the conserved domain (PF04564) hidden Markov model (HMM) shared by U-box from the Pfam 32.0 database (http://pfam.xfam.org/). The protein sequences containing a conserved domain were obtained using the HMMER search tool (http://hmmer.org/) [20], and further SMART analysis was used to verify that four protein sequences without a U-box structure were deleted. Finally, 99 *B. oleracea* U-box genes were identified and the results were consistent with the first method (i.e., retrieval from the BRAD and Bolbase database). At the same time, 60 AtPUB genes homologous to the BoPUB genes were obtained by sequence alignment. Additionally, based on all the genome sequences of *B. oleracea* by transcriptome sequencing in our laboratory, we found all of these genes in the transcriptomes of pollinated *B. oleracea* A4 pistils by BLAST alignment (Table 1).

### 2.3. Phylogenetic, Conservative Motif, and Structure Analysis

**Figure 1:**
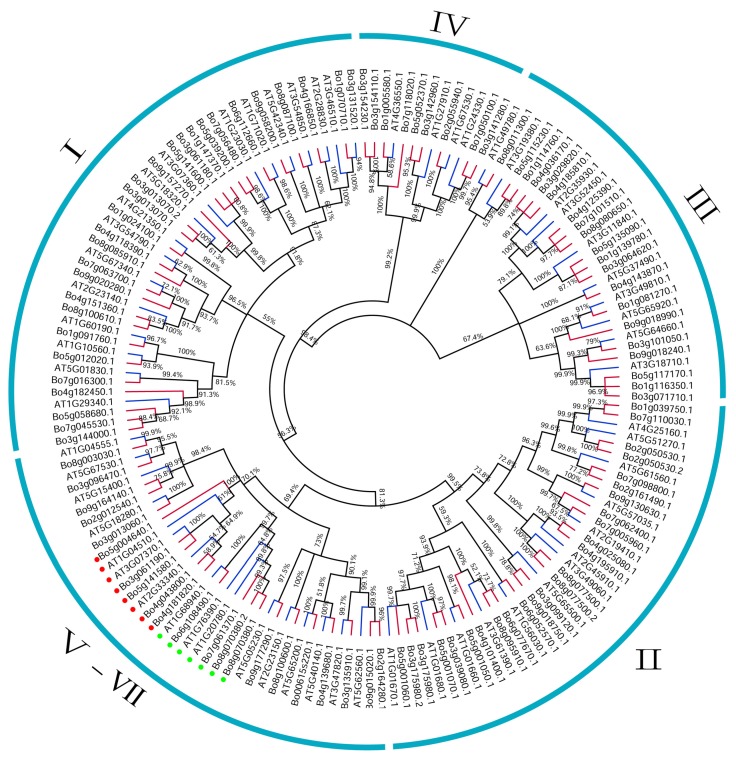
Phylogenetic tree of *B. oleracea* and *A. thaliana* U-box proteins. MEGA X was used to construct the phylogenetic tree with the maximum likelihood (ML) method involving 1000 bootstrap replications. The red lines at the end of the branches indicate the *B. oleracea* gene, the blue lines indicate the *A. thaliana* gene, and the whole tree is divided into seven groups (I–VII). The red dots represent Group VI, the green dots represent Group V, and the remainder represent Group VII. The grouping results of this phylogenetic tree are consistent with those of Figure 3.

The ClutsalW program was used for multiple protein sequence alignments, and MEGA X was used to construct the phylogenetic tree using the maximum likelihood (ML) method with 1000 bootstrap replications [21] (Figure 1). The constructed phylogenetic tree was imported into FigTree software (http://tree.bio.ed.ac.uk/) for presentation. MEME (Multiple EM for Motif Elicitation, http://meme-suite.org/tools/meme) was used to analyze the conserved motifs of proteins online; maximum motifs was set to 15, other parameters were set to defaults, and the analysis results were saved in a MEME.xml format [22]. The genomic-wide gff3 format file of *B. oleracea* was downloaded from EnsemblePlants, and the intron and exon structures of the U-box genome-wide were visualized with GSDS 2.0 (http://gsds.cbi.pku.edu.cn/) [23]. The other domains of the U-box protein were analyzed online using Batch CD-Search (https://www.ncbi.nlm.nih.gov/Structure/bwrpsb/bwrpsb.cgi) and the results were saved in the format of hitdate.txt [24]. The above results were visualized using TBtools [25].

### 2.4. Chromosome Localization, Genome Collinearity, and Ka/Ks Ratio Analysis

**Figure 2:**
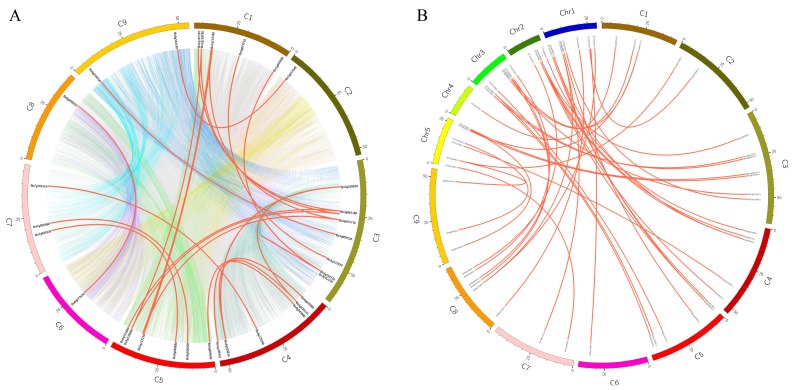
Tandem duplication of the U-box gene in the *B. oleracea* genome and between the *B. oleracea* and *A. thaliana* genomes. (**A**) Tandem replication events between C1–C9 chromosomes of *B. oleracea*. The solid red line indicates the U-box gene replication, and the background with different color blocks indicates all gene duplication of the *B. oleracea* genome. (**B**) Tandem replication of U-box gene between *B. oleracea* C1–C9 and *A. thaliana* Chr1–Chr5. Red solid lines indicate tandem repeats.

Under the Linux system, the chromosome location information of the U-box gene was extracted from the whole genome gff3 file of *B. oleracea* using the Perl language. The chromosome localization and collinearity visualization of the U-box gene were carried out using TBtools and Adobe Acrobat DC (Adobe, Beijing, China).The coding sequence (CDS) of U-box protein was downloaded from the Bolbase and TAIR (https://www.arabidopsis.org/index.jsp) databases, and compared using BLASTALL under the Linux system. The tandem repeat genes were screened according to the standard that the similarity between the two sequences was >80% and the alignment length of the two sequences was greater than 75% of the longer sequences [26]. Collinearity analysis was the completed using Circos software (http://circos.ca/) (Figure 2). The ratio of non-synonymous substitution rate (Ka) to synonymous substitution rate (Ks) of each pair of tandem duplication genes was calculated using KaKs_Calculator2.0 [27].

**Figure 3:**
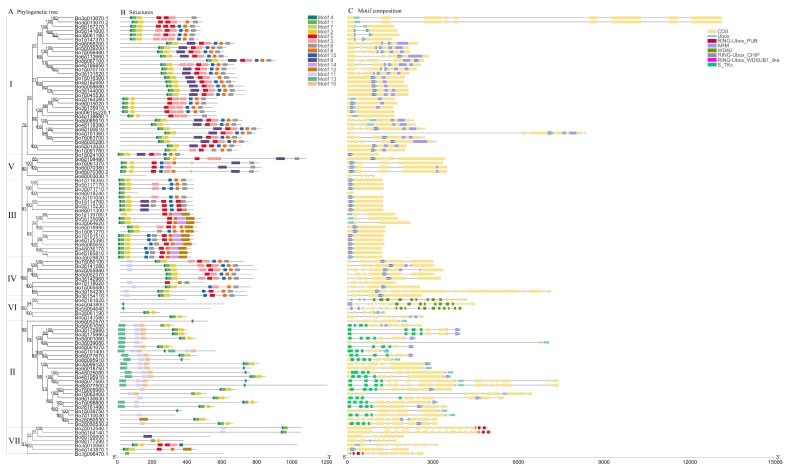
Phylogenetic, conservative motif, and structural analysis of the U-box gene family in *B. oleracea*. (**A**) Phylogenetic tree. MUSCLE was used for coding sequence (CDS) alignment and MEGA X was used to construct the phylogenetic tree with the maximum likelihood (ML) method with 1000 bootstraps; the tree was divided into seven groups (I–VII). (**B**) Conservative motif distribution. The 15 conservative motifs are represented by different colors. See Table 2 for more details. (**C**) Structural distribution. Yellow represents exons, lines represent introns, and other colors represent other structures.

### 2.5. Expression Analysis of U-Box Gene Family and RT-qPCR

Total RNA from the different pollination *B. oleracea* pistil was extracted using an RNA Isolation kit (Tiangen, Beijing, China), and the samples were sent to the Biomarker Technologies Corporation (Beijing, China) for transcriptome sequencing. Using the measured RNA-seq data, including the whole genome information of the U-box in the pistils of *B. oleracea* after 0 min pollination, self-pollination (SP), and cross-pollination (CP) for 15 min, 30 min, and 60 min, the expression levels of these genes were quantified by log_2_(FPKM) values, and the expression results were visualized by TBtools. Fold change ≥2 and FDR <0.01 were used as screening criteria [28]. Thus, 36 differentially expressed genes were screened and identified by DESeq [29], and the expression levels of these genes were then verified by RT-qPCR. Gene-specific primers were designed in the online tool qPrimerDB (https://biodb.swu.edu.cn/qprimerdb/), with BoActin as the internal gene; related RT-qPCR primers are listed in Table S1. RT-qPCR was carried out with a total volume of 20 µL (comprising 10 μL of TB Green Premix Ex TaqTM II, 0.6 μL of each primer, 2 μL of template (×10 diluted complementary DNAs), ROX 0.4 μL, and 6.4 μL of sterile distilled water). Real-time fluorescence quantitative analysis was performed using the Bio-Rad cfx-96 system with the following steps: denaturation at 95 °C for 30 s, followed by 40 cycles of denaturation at 95 °C for 5 s, and annealing/extension at 60 °C for 30 s [30]. Each sample was run through three technical repeats, and the relative expression level of each gene was evaluated using the 2^−ΔΔCt^ algorithm [31].

### 2.6. Promoter Region Analysis of U-Box Differentially Expressed Genes

The 1500-bp sequences upstream of the translation initiation sites of 36 differentially expressed genes were extracted from the *B. oleracea* genome sequence. The cis-acting elements of the promoter region were analyzed and identified with the PLACE database [32]. The cis-acting elements of the promoter region were visualized by GSDS 2.0 (http://gsds.cbi.pku.edu.cn/).

### 2.7. Interaction Network of B. oleracea U-Box Protein

The amino acid sequences of 60 U-box genes in *A. thaliana* were extracted and the interaction network was constructed by the STRING (https://string-db.org/) program [33]. Based on the relationship between orthologous genes of *B. oleracea* and *A. thaliana*, the interaction of the U-box gene was presumed.

### 2.8. Gene Ontology Annotation and KEGG Pathway Enrichment of B. oleracea U-Box Gene

All *B. oleracea* U-box genes were mapped to Gene Ontology (GO, http://www.geneontology.org/) and the GOseq method was selected to calculate the number of genes for each GO term. The KOBAS online database was used to analyze the metabolic and signal transduction pathways involved in the U-box gene [34], and the Kyoto Encyclopedia of Genes and Genomes (KEGG, https://www.genome.jp/kegg/) was then used to verify its specific metabolic pathways.

## 3. Results and Discussion

### 3.1. Identification, Classification, and Phylogenetic Analysis of U-Box Gene Family

We used two methods (retrieval from the BRAD and Bolbase databases, and the HMMER search tool) to retrieve and identify members of the *B. oleracea* U-box gene family; the results were consistent. Four protein sequences (Bol019081, Bol018658, Bol011584, and Bol045810) that clearly did not contain the U-box structure were deleted, and finally 99 *B. oleracea* U-box genes were screened (Table 1). At the same time, 60 AtPUB genes homologous to BoPUB were obtained (Table 1), in which AT3G10370.1 and AT3G10370.2 indicate different transcripts of the *A. thaliana* AtPUB gene. Based on the results of transcriptome sequencing analysis in our laboratory, we matched the retrieved BoPUB genes with the transcriptome sequencing gene by sequence alignment and further confirmed that they were consistent (Table 1). In addition, we retained the four transcriptome genes without a U-box structure excluded above for subsequent analyses.

Azevedo et al. clustered PUBs from *A. thaliana* based on conserved domains [35]. However, the domains of the 99 PUB genes in *B. oleracea* are somewhat complicated, so we considered combining phylogenetic trees and domains as a basis for grouping. According to the evolutionary relationship of the phylogenetic tree, conserved motif, gene structure, etc., the retrieved BoPUB genes were divided into seven groups (I–VII), and named BoPUB 1–BoPUB 99 (Table 1). The data showed that most *A. thaliana* genes have three homologous genes in *B. oleracea*, such as AtPUB5, 9, 22, 24, 29, 36, 46, and 56. AtPUB17 has four homologous genes in *B. oleracea*. This indicates that the U-box gene expansion and evolution between *B. oleracea* and *A. thaliana* are closely related.

In order to better understand and verify the phylogenetic relationships between members of the U-box gene family in *B. oleracea* and *A. thaliana*, 103 BoU-box and 60 AtU-box genes were constructed using MEGA X software with the maximum likelihood method and divided into seven groups (Figure 1). The classification results were consistent with those in Table 1. Groups I, II, III, and IV of *B. oleracea* genes in Figure 3A are also clustered together in Figure 1 and included in each group. The clustering results of Figure 1 are consistent with those shown in Figure 3A; the slight difference is that the three groups (V, VI, and VII) come together to form a large branch (Figure 1). This indicates that the classification and naming of members of the *B. oleracea* U-box gene family in Table 1 are reliable. Careful observation of Figure 1 shows that each *A. thaliana* U-box gene produces at least one copy of the *B. oleracea* U-box gene and up to four copies.

Compared with the study by Wang et al. [10]. The largest group consists of proteins containing the ARM domain (I), but in different numbers (41 proteins in *B. rapa* and 28 proteins in *B. oleracea*). We classify proteins containing a large number of S_TKC (N-terminal domain of the eukaryotic serine threonine kinase) domains into a second broad category (II), and almost all U-box gene domains in *B. oleracea* contain a RING-finger. It is worth noting that Wang et al. identified two WD40-containing proteins from the evolution of *AT1G04510.1* and *AT2G33340.1* in *B. rapa*. However, three WD40 repeats were identified in *B. oleracea*, and *AT2G33340.1* evolved to produce two WD repeats (VI). Additionally, AT3G07370.1 evolved a protein containing the TPR domain in *B. rapa*, while two proteins containing the TPR domain were produced in *B. oleracea* (VI).

### 3.2. Collinear Analysis within the Genome of B. oleracea and between the Genomes of B. oleracea and A. thaliana

In order to reveal the gene duplication of the U-box family members in *B. oleracea*, the genome differences between *B. oleracea* and *A. thaliana* were analyzed using the Circos software and the Perl language under the Linux system to complete collinearity analysis. Figure 2A is an intra-genomic collinearity analysis map based on the location information of nine chromosomes in the *B. oleracea* genome and the selected 24 pairs of tandem repeat genes; the red line indicates the collinearity U-box gene in the genome and the different colors of the background are collinear blocks from collinear gene pairs of the entire *B. oleracea* genome. Figure 2B shows 39 pairs of tandem repeats between *B. oleracea* and *A. thaliana*, and the red line indicates the collinearity of the U-box gene between these two genomes. Schedule S-2 provides detailed information on tandem duplication genes (Table S2).

The results of collinear analysis indicated that both orthologous copies and paralogous copies were present between *B. oleracea* and *A. thaliana*. *Bo1g005580.1*, *Bo3g064620.1*, *Bo3g154110.1*, *Bo4g036170.1*, and *Bo5g117170.1* each have two paralogous copies in the *B. oleracea* genome (Figure 2A). These paralogous copies may also be an important factor in the occurrence of gene clusters and diversity distribution in the U-box family of *B. oleracea* [36]. Figure 2B shows that multiple AtPUB genes (*AT1G04510.1*, *AT1G20780.1*, *AT2G33340.1*, *AT3G11840.1*, *AT3G46510.1*, *AT5G1540.1*, and *AT5G65920.1*) in *A. thaliana* have two orthologous copies, and the *AT2G35930.1* gene has three orthologous copies in *B. oleracea*. Although these orthologous copies are located in different species (*B. oleracea* and *A. thaliana*), the BoPUB gene was derived from a common ancestor shared with *A. thaliana*, so their functions are generally similar. In addition to these polygene duplications, there are a large number of single gene duplications. Although these single gene duplications often result in gene loss [37], the final retained genes are of great significance in evolution [38]. In short, extensive whole genome duplication (WGD) is considered to be the main driver of species diversity and the primary mechanism for acquiring new genes [39,40].

The vast majority of gene duplicates may be silenced over time, but a small number of genes purifying selection remain. Although these may rarely evolve new functions, the stochastic silencing of such genes also plays an important role in the origin of new species [41]. To gain a deeper understanding of the selection of these genes after replication, we calculated the ratio of the non-synonymous substitution rate (Ka) to the synonymous substitution rate (Ks) (Table S3-1 and Table S3-2). It was found that the Ka/Ks value of 24 pairs of tandem genes in the genome ranged from 0.09 to 0.65; the Ka/Ks value of 39 pairs of tandem genes between genomes ranged from 0.04 to 0.50; and all Ka/Ks values were less than 1, indicating that members of the U-box family in the *B. oleracea* genome and between the *B. oleracea* and *A. thaliana* genomes underwent purified selection during long-term evolution [42]. Gene duplication events are very important for studying the evolutionary mechanisms of plant genes, and tandem repeats have a significant impact on the amplification and evolution of gene families in plant genomes [43].

### 3.3. Phylogenetic, Conservative Motif, and Structural Analysis of the U-Box Gene Family in B. oleracea

Firstly, a phylogenetic tree (Figure 3A) was constructed using MEGA X software with the ML method based on the CDS of all *B. oleracea* U-box genes. Secondly, 15 conserved motifs of the BoU-box family members were identified by MEME online analysis (Figure 3B), and varied from 8 to 50 amino acids in length. Details of 15 presumed conserved motifs are shown in Table 2. Batch CD-Search was used to analyze the gene structure online (Figure 3C). From comprehensive analysis of this information, it was finally determined that the 103 U-box genes could be roughly divided into the I–VII groups. Additionally, four genes (*Bo8g003030.1*, *Bo9g018240.1*, *Bo7g118020.1*, and *Bo8g100600.1*) were found that obviously lacked a U-box domain in the analysis of the gene structure. UniProt (https://www.uniprot.org/) online analysis confirmed this result.

In general, U-box proteins that are clustered in the same type have similar conserved motifs, indicating that these proteins have similar functions. In combination with Figure 3A,B, the U-box protein motif in the same group of phylogenetic trees is highly similar and conserved, and almost all of them have Motif 4, 1, 7, and 2 (except for Group VII); this indicates that these four motifs play an important role in the BoU-box gene family (Figure 3B). Referring to EnsemblePlants and UniProt databases, the structure information of the U-box gene family in *B. oleracea* was determined (Figure 3C), including CDS (exon), Ubox, RING-Ubox_PUB, Arm, WD40, RING-Ubox_WDSUB1_like, S_TKc, and RING-Ubox_CHIP. According to the existing research findings, the ligase (E3) recruits E2 and the target protein is the last step of ubiquitin (Ub) transfer to the substrate. These three types of E3 enzymes are: HECT (homologous to E6-AP C-terminus), RING (Really Interesting New Gene), and U-box [35]. Protein interaction modules (e.g., WD40 repeats, TPR, and ARM repeat domains) are involved when U-box and RING act as scaffold molecules to recruit and localize substrates [44,45]. Therefore, the domain of the U-box family, contains many RING-finger and U-box domains, which indicates that the U-box family gene of *B. oleracea* has abundant E3 ligase characteristics. This conclusion is demonstrated in the published work of Trujillo et al. [6].

According to the NCBI query, it is clear that RING-Ubox_CHIP, RING-Ubox_PUB, and RING-Ubox_WDSUB1_like belong to the RING_Ubox Superfamily. RING_Ubox contains the RING-finger and U-box domains. RING-finger is a special type of zinc finger with 40 to 60 residues. The U-box is a modified form of the RING-finger domain, lacking metal chelate cysteine and histidine. This consists of three β-sheets and an α-helix, which mainly relied on salt and hydrogen bonds to stabilize their structures, rather than chelated metal ions [46,47]. The results of the graph (Figure 3C) indicate that almost all of the identified genes contain the U-box and RING-Ubox_PUB domains, and the U-box domain coincides with the RING-Ubox_CHIP/WDSUB1_like PUB, suggesting that they may have synergistic effects. RING-Ubox_PUB is a common PUB protein in plants, also known as U-box domain protein. Most of the family’s AtPUB is called PUB protein containing the ARM domain, a type of improved RING-finger, which is not only related to the regulation of cell death and defense, but also plays an important role in other plant-specific pathways, such as controlling self-incompatibility and acting in abiotic stress. The two genes (*Bo3g061190.1* and *Bo5g141580.1*) containing the RING-Ubox_CHIP domain belong to the Group VI, which is homologous to *A. thaliana* AtCHIP (*AT3G07370.1*) from the protein interaction network, indicating that these three genes may be functionally similar. The RING-Ubox_WDSUB1_like structure exists in Group II. WDSUB1 is a non-characterized protein containing seven WD40 repeats and one SAM domain in addition to the U-box. Group VI has three genes in the WD40 domain, and the WD-40 repetitive sequence, also known as the WD or β-transferrin repeat, is a motif with approximately 40 amino acids that normally terminates in the Trp-Asp dipeptide [48]. WD40-repeat proteins are a large family found in all eukaryotes and are involved in a variety of functions, including signal transduction, transcriptional regulation, and apoptosis [49]. Several WD40-containing proteins in *A. thaliana* are key regulators of plant-specific developmental events. Group II also has an N-terminal domain of the eukaryotic serine threonine kinase, S_TKc. Protein kinases are enzymes belonging to a very broad protein family that play a role in a variety of cellular processes, including division, proliferation, apoptosis, and differentiation. They have a common conserved catalysis, and homology of the N-terminal domain with the USP family indicates that the N-terminal domain is involved in ATP binding [50,51,52].

In addition, there are many proteins with ARM and RING-Ubox domains in groups I, V, and VI, which have the same domain as ARC1 and belong to the PUB-ARM Superfamily. ARM repeats are characteristic repetitive amino acid sequences with a length of approximately 40 residues found in proteins, and proteins containing these sequences typically contain several tandem repeat copies [53,54]. Studies have shown that the interaction between the C-terminal arm repeat region ARM of the ARC1 protein with functional E3 ubiquitin ligase and the S-locus receptor kinase domain is part of the signal transduction pathway, leading to self-incompatibility pollen rejection [2,55]. It has been suggested that the *A. thaliana* ARM repeat protein is a member of the U-box E3 ubiquitin ligase family, and AtPUB-ARM can interact with the target substrate through its ARM repeat or UND (the N-terminal region of U-box). Samuel et al., using AtPUB13, 14, 45 (similar to ARC1 structure, containing UND, U-box, and ARM domain) and S-locus receptor kinase (SRK) for yeast double-hybrid experiments, proved that they can interact well [18], but whether all genes with UND structure participate in SI signal transduction still needs further study. Mudgil et al., identified 17 PUB genes with an UND structure through protein phylogenetic analysis of *A. thaliana* [56]; whether the homologous genes of these PUB genes in *B. oleracea* also have an UND structure also needs to be further studied. In addition, not all AtPUB-ARMs contain UND. In vitro ubiquitination assays also indicate that UND only promotes binding to ARM repeat regions and does not affect E3 ligase activity, so the AtPUB-ARM family may regulate the substrate due to the diversity of the ARM repeat region, thus controlling a series of different cellular processes [56]. Thus, it can be seen that the specific function of the UND domain in self-incompatibility needs further research. Trujillo proposed that in order to have a deeper understanding of the function of PUB, structural analysis would be an important means of understanding the complex interactions between PUB domains, especially for the unique PUB composed of UND, U-box, and ARM repeats [6]. It is hoped that the structural analysis of this section will provide valuable information for the in-depth study of the PUB gene in *B. oleracea*.

### 3.4. Chromosome Localization of B. oleracea U-Box Gene

**Figure 4:**
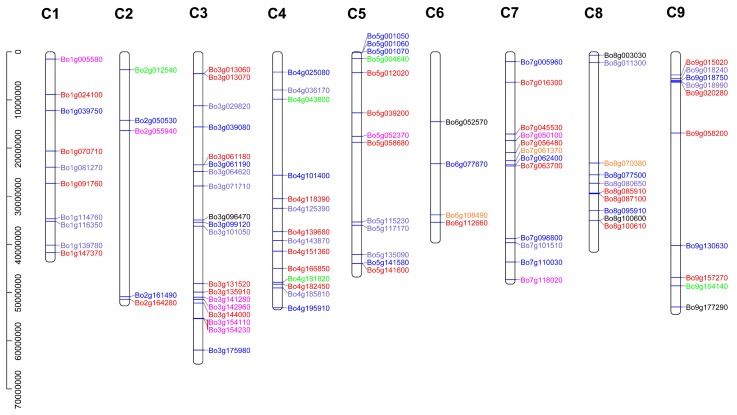
Localization of the U-box gene on chromosomes. Different colors represent differently grouped U-box genes.

As shown in Figure 4, a total of 102 BoPUB genes are mapped to C1–C9 chromosomes. The *Bo00615s220.1* gene is a novel gene identified by transcriptome sequencing, and is located on the chromosome scaffold and cannot be visualized. Different groups of genes are represented by different colors according to the aforementioned grouping. The number of genes on each chromosome is unevenly distributed, and up to 21 genes are distributed on the C3 chromosome (Figure 4), indicating the complex diversity of the U-box family genes in *B. oleracea*. In addition, there are more gene clusters in C3, C4, C5, C7, C8, and C9 chromosomes, indicating that these gene amplification products belong to a common ancestor. The homologous gene relationship between C3, C1, and C5 is the most obvious, since there are many tandem duplication events (Figure 2A and Table S2).

### 3.5. Interaction Network of U-Box Protein

**Figure 5:**
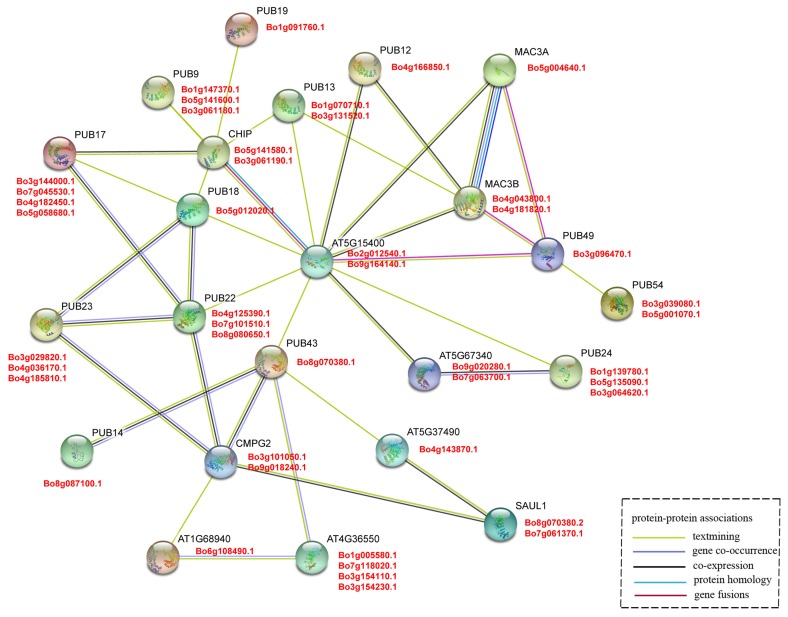
Interaction network of U-box proteins. The red font indicates *B. oleracea* and the black font indicates *A. thaliana*.

To analyze the protein interactions between the U-box genes in *B. oleracea*, a protein interaction network was constructed with 60 orthologous genes in *A. thaliana* using the STRING software (Figure 5). Each *A. thaliana* gene corresponds to several orthologous *B. oleracea* genes, and a total of 23 *A. thaliana* genes and 45 *B. oleracea* genes are involved in the interaction network. These genes have catalytic activity and are related to the ubiquitination of proteins. The central node gene (*AT5G15400*) is Mutant snc1-enhancing 3 (MUSE3), an E4 ubiquitin ligase involved in polyubiquitination of NLRs, which seems to function downstream of CPR1 to facilitate the polyubiquitination and degradation of SNC1 and RPS2 [57]. In addition, the MUSE3 ortholog yeast E4 ubiquitin ligase UFD2 can promote the extension of ubiquitin chains [58]. This indicates that MUSE3 plays an important role in the ubiquitination pathway of proteins. *Bo2g012540.1* and *Bo9g164140.1* are the orthologous genes of MUSE3, and the evolutionary relationship is close and the gene structure is similar (Figure 3, Group VII), so it is likely that both have similar functions to MUSE3.

In this network, AtCHIP (C-terminal of Hsp70 interacting protein) contains a U-box domain and three tetratricopeptide repeats (TPR) domain that mediates its interaction with Hsp90 and Hsp70 chaperones. As an E3 ubiquitin ligase of the protein phosphatase 2A subunit, it can alter the response of plants to abscisic acid treatment and can also play a key role under temperature stress conditions [59,60,61]. Through the interaction network, it can reliably reveal the homology, co-occurrence, and co-expression of *B. oleracea* and *A. thaliana* U-box gene. Several genes (AtCHIP, AtPUB 17/18/22/23/43/14, and CMPG2) formed a relatively close interaction and showed homology and co-expression (Figure 5). Among these, the interaction of AtPUB18/ 22/ 23 can participate in a variety of biological processes. AtPUB18 may mediate EXO70B1 ubiquitination and participate in the regulation of ABA-mediated stomatal movement, and AtPUB22 and AtPUB23 coordinate control of drought signaling pathways [62,63]. The above results indicate that a variety of AtPUB proteins can individually affect or interact with each other to regulate related proteins in different signaling pathways, which provides a strong rationale for the study of related pathways involved in the BoPUB genes.

### 3.6. Gene Ontology Annotation and KEGG Pathway Enrichment Analysis of U-Box genes in B. oleracea

**Figure 6:**
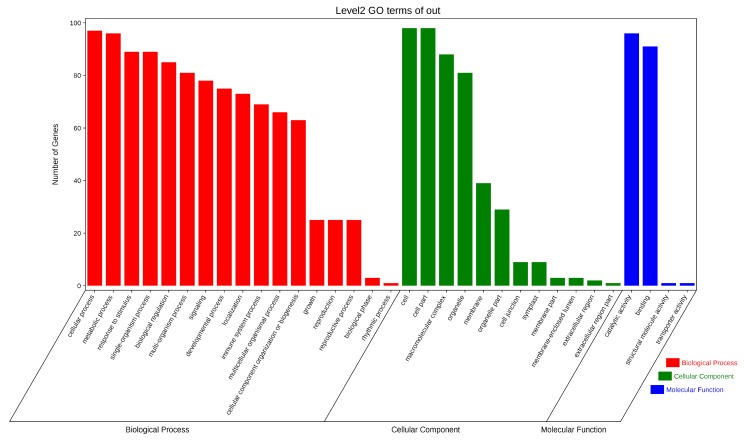
Gene Ontology enrichment analysis of the U-box gene in *B. oleracea*. Red indicates biological processes, green represents cell components and blue indicates molecular function. The ordinates represent the number of genes enriched into the entry.

After studying the interaction of U-box proteins, we continued the GO annotation and KEGG pathway enrichment analysis to gain a deeper understanding and validation of the biological functions of these genes (Figure 6, Table S4, and Table 3). The results showed that a total of 1689 GO terms were significantly enriched, including biological processes, cell components, and molecular functions (Figure 6). Among these, biological processes are enriched with the most items, mainly cell processes, metabolic regulation, stimulation reactions, signal transmission, etc. The most common cellular components include cells, organelles, and cell membranes. The most abundant molecular functions are catalytic activity and molecular binding. The GO annotation is consistent with the results of the protein interaction network analysis (Table S4 and Figure 5).

The metabolic or signal transduction pathways involved in U-box genes were analyzed online using the KOBAS database, and the specific metabolic pathways were verified by KEGG. It was found that some genes are mainly involved in three pathways: ubiquitin-mediated proteolysis, splicing, and protein processing in the endoplasmic reticulum (Table 3).

### 3.7. Expression Analysis and RT-qPCR

**Figure 7:**
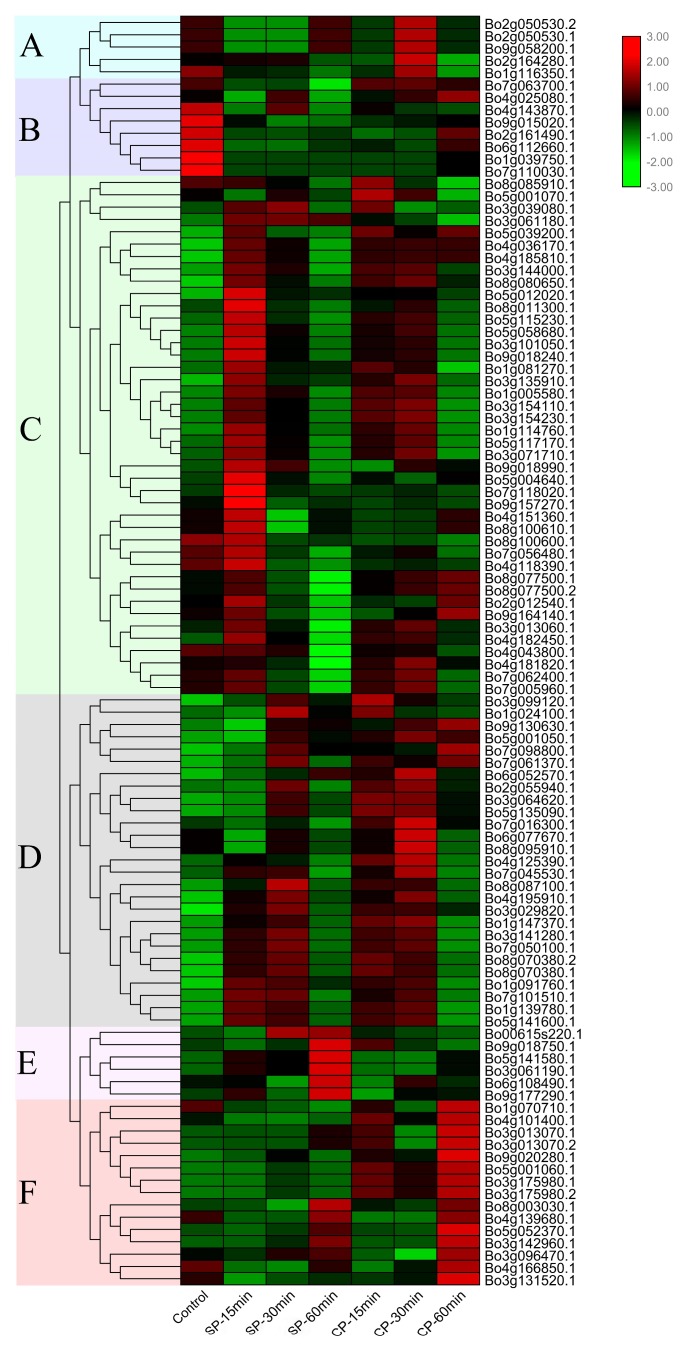
Expression pattern of the U-box gene after different pollination conditions and times. The FPKM values were obtained from RNA-seq and normalized by log_2_ (Table S5), and the color code represents the transformation value of log_2_(FPKM). Bright green represents low expression and dark red represents high expression. According to the trend of gene expression in different pollination conditions and times, the heat map is divided into six groups (**A**–**F**).

In order to understand the expression of the U-box whole gene family members of *B. oleracea* in different pollination conditions and times, the expression levels of these 103 genes were quantified by FPKM (fragments per kilobase of transcript per million fragments mapped) values of RNA-seq data [64]. The heat map was drawn according to the log_2_ (FPKM) values (Table S5) of pollinated 0 min, CP-15/30/60 min, and SP-15/30/60 min, and clustered into A–F based on the expression profile characteristics of these genes (Figure 7 and Table S5).

The five genes contained were significantly up-regulated after CP for 30 min (Figure 7A). As show in Figure 7B, with the exception of Bo4g025080.1, which was expressed at a higher level after CP-60 min, most were expressed at 0 min pollination. Figure 7C shows that most genes were significantly expressed after SP-15 min, and decreased after 30 min and 60 min, and weakly expressed at each time point under the condition of CP. Most of the genes were highly expressed after SP-15 and SP-30 min or CP-15 and CP-30 min, but the expression level was extremely low at 0 min pollination (Figure 7D). The results of Figure 7E,F are more intuitive; the genes in Figure 7E were significantly expressed at SP-60 min, while the expression in Figure 7F is extremely high after CP-60 min.

**Figure 8:**
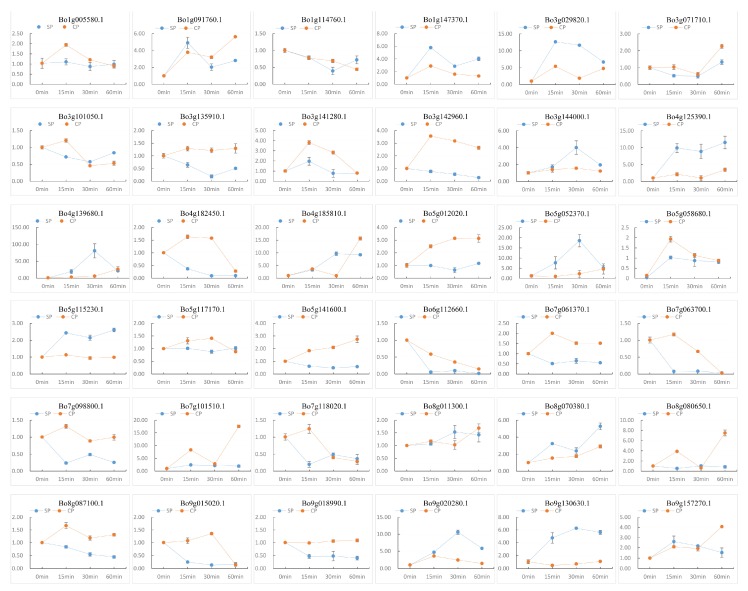
RT-qPCR detection of DEG expression levels. The 36 differentially expressed genes selected from the RNA-seq data were detected by RT-qPCR to verify the expression of the U-box gene in pollination at 0 min, CP-15/30/60 min, and SP-15/30/60 min. The abscissa indicates the time after pollination, the ordinate indicates the relative expression level, and the error bars indicate the standard deviation (±SD) of the three biological replicates under different pollination conditions.

Gene expression is temporally and spatially specific. Genes with significant differences in expression levels under different conditions are called differentially expressed genes (DEG). A total of 36 differentially expressed genes were screened out using fold-change ≥2 and FDR <0.01 as screening criteria in the process of differentially expressed gene detection [29]. The expression levels of 36 differentially expressed genes were detected by RT-qPCR and are plotted as a line graph to verify the up- and -down-regulation of pollination at 0 min, CP-15/30/60 min, and SP-15/30/60 min (Figure 8 and Table S1). The abscissa in Figure 8 indicates the time after pollination, the ordinate indicates the relative expression level, and the error bars indicate the standard deviation (±SD) of the three biological replicates under different pollination conditions. RT-qPCR results showed that some genes were significantly up-regulated after SP-15 min or SP-30 min, such as *Bo1g147370.1*, *Bo3g029820.1*, *Bo4g125390.1*, *Bo5g052370.1*, and *Bo9g130630.1*. It is notable that the up-regulation of these genes under the condition of CP is not obvious, indicating that these genes are highly likely to be involved in the SI-related signaling transduction pathways in *B. oleracea*. We further analyzed the relationship between these genes and the results of bioinformatics analysis, and found that the conserved motifs and structures of these genes are similar (Figure 3). Additionally, *Bo1g147370.1*, *Bo3g029820.1*, and *Bo4g125390.1* formed a relatively close interaction and showed homology and co-expression (Figure 5). These proteins have ubiquitin-protein transferase activities [56]. *Bo1g147370.1* may be involved in the abscisic acid-mediated signaling pathway [18], while *Bo3g029820.1* and *Bo4g125390.1* coordinate control of drought signaling pathways [62]. Interestingly, from the chromosomal localization of highly significant differentially expressed genes (Figure 4), such genes are mostly located at C1, C3, and C5. Combined with tandem replication events (Figure 2A and Table S2), there is significant gene duplication among the three chromosomes. Whether there is a connection between the genes on these chromosomes is worthy of consideration.

### 3.8. Cis-Regulatory Element Analysis in the Promoter Regions of U-Box Differentially Expressed Genes

**Figure 9:**
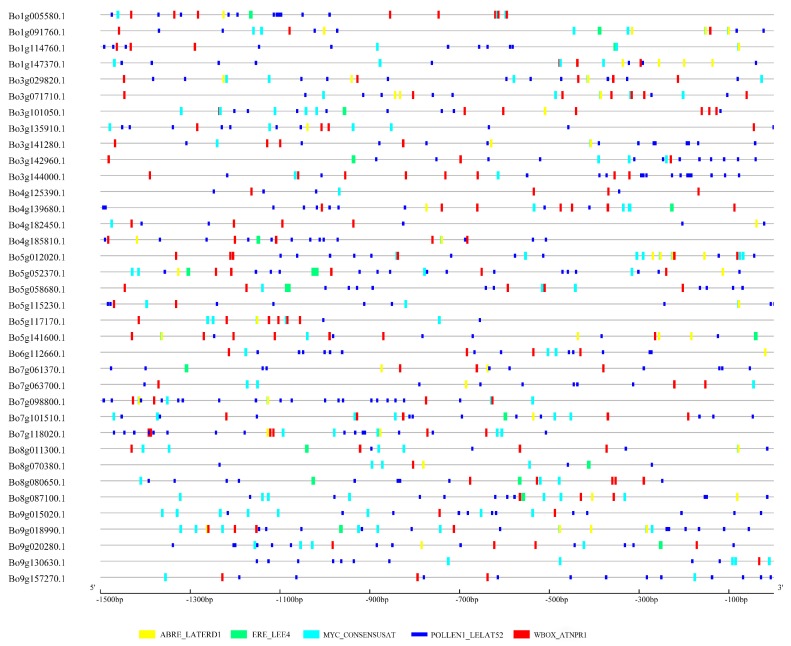
Cis-regulatory element analysis in the promoter regions. ABRE_LATERD1: abscisic acid reaction element; ERE_LEE4: ethylene reaction element; MYC_CONSENSUSAT: dehydration reaction and abscisic acid signaling related reaction element in *A. thaliana*; POLLEN1LELAT52: pollen-specific cis-acting element; WBOX_ATNPR1: pressure and defense-related response element.

To further elucidate the differential expression results of 36 genes and their putative role in the pollination process, PLACE and GSDS 2.0 were used to analyze and visualize the cis-regulatory elements in the promoter region [32]. The main regulatory elements include: hormone-related regulatory elements (ABRE, ERE), pollen-specific cis-regulators (POLLEN_lat52), dehydration-related action elements (MYC), and pressure and defense related regulatory elements (W-box) (Figure 9 and Table S6).

The ABRE regulatory element can transcriptionally regulate the abscisic acid (ABA) response gene [65], and a novel cis-acting element ABRE_ERD1 has been shown to be up-regulated in response to water stress and yellowing in *A. thaliana*, indicating that EDR1 elements play an important role in dehydration induction and stress [66]. MYC_CONSENSUSAT is the promoter region recognition site of the dehydration reaction gene rd22 in *A. thaliana*, and MYC is a cis-acting element in drought-induced rd22 gene expression [67]. Follow-up studies have shown that the AtMYC2 protein plays the role of transcriptional activator in ABA inducible gene expression under plant drought stress [68]. These differentially expressed U-box genes contain a large number of pollen-specific cis-acting elements of POLLEN1LELAT52 (AGAAA) (Figure 9). Studies have shown that this regulatory element is involved in the precise pollen development and tissue-specific expression of tomato lat52 and can affect the transcriptional activation of lat52 [69]. LeMAN5 endo-β-mannanase, which is related to pollen germination and pollen tube elongation in tomato, has a 5’-upstream region containing four copies of POLLEN1LELAT52, which can be used for pollen fertility control [70]. “W-box” found in promoter of *A. thaliana* NPR1 gene, they were recognized specifically by salicylic acid (SA)-induced WRKY DNA binding proteins. WRKY proteins are DNA-binding proteins that recognize the W-box elements found in the promoters of a large number of plant defense-related genes [71]. *Arabidopsis* transcription factor AtWRKY18 enhances developmental regulation of plant defense responses [72]. Analysis of the above cis-acting elements indicated that these differentially expressed *B. oleracea* U-box genes can not only respond to different biotic or abiotic stresses, but are also closely related to the pollen development of *B. oleracea*.

## 4. Conclusions

In this study, four PUB family genes without U-box characteristics were excluded from the *B. oleracea* genome database, and a total of 99 BoPUB genes were identified. Through the comprehensive analysis of the phylogeny, conservative motifs, and gene structure of the BoPUB genes, verified by their homologous evolutionary relationship and structure of the AtPUB gene, these genes were grouped and named. At the same time, the evolutionary relationship, gene expansion, and functional diversity of the PUB gene family in *B. oleracea* were also revealed. Chromosome localization and collinear analysis showed that members of the U-box family were purified and selected during long-term evolution, and these genes may be products of amplification from the same ancestral gene. Protein interaction network analysis indicated that the BoPUB protein is widely involved in the ubiquitination pathway and regulates downstream proteins, which was verified by GO annotation and KEGG pathway enrichment analysis. We analyzed the expression characteristics of the BoPUB genes under different pollination conditions, and the related genes in the U-box gene family of *B. oleracea* likely to be involved in SI reaction were identified by RT-qPCR. A large number of cis-acting elements related to pollen specificity, hormone regulation, and dehydration reactions were found in the promoters of differentially expressed genes, which further proved that these genes not only play an important role in the regulation of plant development and abiotic stress, but are also involved in pollination. These genes should be selected for follow-up study of SI reaction in *B. oleracea*. This study not only systematically analyzed the characteristics and evolution of the U-box family of *B. oleracea*, but also provided candidate genes for SI reaction and signal transduction in *B. oleracea*, thereby providing a reference for revealing how U-box genes participate in SI.

## Figures and Tables

**Table 1:** The U-box Genome-wide information in *Brassica oleracea*.

Group	*B. oleracea* Gene	BoPUB ID	Chr	Genomic Location	AtPUB ID	*A. thaliana* Gene	E-Value	Identity (%)	Transcriptome ID	E-Value	Identity (%)
**I**	*Bol019224*	BoPUB1	C09	4,934,468–4,937,484	AtPUB2	*AT5G67340.1*	0	84.53%	Bo9g020280.1	0	98.56%
	*Bol014311*	BoPUB2	Scaffold000175_P1	442,705–445,437	AtPUB2	*AT5G67340.1*	0	78.79%	Bo7g063700.1	0	99.19%
	*Bol044176*	BoPUB3	C04	24,020,183–24,022,584	AtPUB3	*AT3G54790.1*	0	90.02%	Bo4g118390.1	7.97 × 10^−133^	100.00%
	*Bol008980*	BoPUB4	Scaffold000240	145,239–147,580	AtPUB3	*AT3G54790.1*	0	88.12%	Bo8g085910.1	0	100.00%
	*Bol045811*	BoPUB5	C08	34,875,756–34,878,494	AtPUB4	*AT2G23140.1*	0	86.94%	Bo8g100610.1	0	100.00%
	*Bol031706*	BoPUB6	Scaffold000053	637,493–643,779	AtPUB4	*AT2G23140.1*	0	87.92%	Bo4g151360.1	2.58 × 10^−108^	99.64%
	*Bol028412*	BoPUB7	C01	7,866,975–7,868,078	AtPUB8	*AT4G21350.1*	2 x 10^−^^149^	78.81%	Bo1g024100.1	0	98.11%
	*Bol008152*	BoPUB8	C01	38,471,230–38,472,813	AtPUB9	*AT3G07360.1*	0	84.52%	Bo1g147370.1	0	100.00%
	*Bol007667*	BoPUB9	C05	32,165,369–32,167,119	AtPUB9	*AT3G07360.1*	0	85.00%	Bo5g141600.1	0	100.00%
	*Bol033996*	BoPUB10	Scaffold000040	700,223–702,053	AtPUB9	*AT3G07360.1*	0	85.54%	Bo3g061180.1	0	100.00%
	*Bol016165*	BoPUB11	C07	4,511,321–4,514,149	AtPUB10	*AT1G71020.1*	0	80.22%	Bo6g112660.1	0	98.50%
	*Bol012315*	BoPUB12	C06	23,724,580–23,726,780	AtPUB11	*AT1G23030.1*	0	84.00%	Bo7g056480.1	0	97.94%
	*Bol023511*	BoPUB13	Scaffold000099_P1	524,243–526,409	AtPUB11	*AT1G23030.1*	0	86.88%	Bo5g039200.1	0	99.73%
	*Bol033248*	BoPUB14	C04	32,033,767–32,036,354	AtPUB12	*AT2G28830.1*	0	81.45%	Bo4g166850.1	0	100.00%
	*Bol018774*	BoPUB15	C01	15,040,202–15,042,653	AtPUB13	*AT3G46510.1*	0	88.01%	Bo1g070710.1	0	100.00%
	*Bol041392*	BoPUB16	C03	35,763,128–35,765,315	AtPUB13	*AT3G46510.1*	0	86.17%	Bo3g131520.1	0	100.00%
	*Bol045384*	BoPUB17	C08	29,129,169–29,134,639	AtPUB14	*AT3G54850.1*	0	85.61%	Bo8g087100.1	0	98.26%
	*Bol035302*	BoPUB18	Scaffold000035_P2	510,161–512,658	AtPUB15	*AT5G42340.1*	0	85.12%	Bo9g058200.1	0	99.39%
	*Bol000236*	BoPUB19	Scaffold000960	766–2784	AtPUB16	*AT5G01830.1*	0	81.20%	Bo7g016300.1	0	98.96%
	*Bol008579*	BoPUB20	C03	44,740,354–44,742,513	AtPUB17	*AT1G29340.1*	0	81.53%	Bo3g144000.1	0	99.31%
	*Bol037877*	BoPUB21	C04	35,662,808–35,664,799	AtPUB17	*AT1G29340.1*	0	75.45%	Bo4g182450.1	0	99.00%
	*Bol041877*	BoPUB22	C06	17,530,961–17,533,096	AtPUB17	*AT1G29340.1*	0	80.70%	Bo7g045530.1	0	99.63%
	*Bol001099*	BoPUB23	Scaffold000469	39,061–41,190	AtPUB17	*AT1G29340.1*	0	76.53%	Bo5g058680.1	0	99.62%
	*Bol036718*	BoPUB24	C05	25,576,732–25,578,822	AtPUB18	*AT1G10560.1*	0	81.99%	Bo5g012020.1	0	100.00%
	*Bol036591*	BoPUB25	C01	22,029,097–22,031,136	AtPUB19	*AT1G60190.1*	0	79.36%	Bo1g091760.1	0	98.77%
	*Bol008036*	BoPUB26	C02	41,702,893–41,704,551	AtPUB38	*AT5G65200.1*	0	78.67%	Bo00615s220.1	0	98.19%
	*Bol024918*	BoPUB27	C03	39,891,535–39,893,145	AtPUB39	*AT3G47820.1*	0	84.62%	Bo3g135910.1	0	100.00%
	*Bol012875*	BoPUB28	Scaffold000192	121,054–122,718	AtPUB40	*AT5G40140.1*	0	82.37%	Bo4g139680.1	0	97.12%
	*Bol020697*	BoPUB29	Scaffold000121_P2	233,911–235,575	AtPUB41	*AT5G62560.1*	0	80.55%	Bo2g164280.1	0	98.69%
	*Bol019195*	BoPUB30	Scaffold000133	1,178,856–1,180,623	AtPUB41	*AT5G62560.1*	0	81.64%	Bo9g015020.1	0	99.46%
	*Bol034489*	BoPUB31	C03	3,652,498–3,654,286	AtPUB46	*AT5G18320.1*	4 × 10^−131^	82.50%	Bo3g013070.1	0	98.23%
	*Bol034491*	BoPUB32	C03	3,671,228–3,673,202	AtPUB46	*AT5G18320.1*	9 x 10^−131^	78.61%	Bo3g013070.2	0	89.87%
	*Bol019726*	BoPUB33	C09	31,567,100–31,568,692	AtPUB46	*AT5G18320.1*	1 × 10^−151^	78.28	Bo9g157270.1	0	100.00%
**II**	*Bol037390*	BoPUB34	C08	24,303,890–24,311,884	AtPUB32	*AT3G49060.1*	0	90.48%	Bo8g077500.2	3.98 × 10^−98^	99.50%
	*Bol003480*	BoPUB35	Scaffold000349	333,337–337,418	AtPUB32	*AT3G49060.1*	0	87.64%	Bo8g077500.1	0	89.62%
	*Bol030222*	BoPUB36	C04	3,124,409–3,127,799	AtPUB33	*AT2G45910.1*	0	86.31%	Bo4g025080.1	0	100.00%
	*Bol021760*	BoPUB37	C04	40,486,202–40,489,850	AtPUB33	*AT2G45910.1*	0	86.82%	Bo4g195910.1	0	98.80%
	*Bol022718*	BoPUB38	C06	7,979,725–7,989,744	AtPUB34	*AT2G19410.1*	0	85.09%	Bo7g005960.1	0	99.47%
	*Bol003293*	BoPUB39	Scaffold000357	194,515–201,007	AtPUB34	*AT2G19410.1*	4 × 10^−130^	79.34%	Bo7g062400.1	7.53 × 10^−107^	100.00%
	*Bol039498*	BoPUB40	C01	11,366,737–11,368,105	AtPUB35	*AT4G25160.1*	8 × 10^−172^	87.19%	Bo1g039750.1	0	100.00%
	*Bol042214*	BoPUB41	C06	43,741,380–43,743,553	AtPUB35	*AT4G25160.1*	5 × 10^−92^	97.06%	Bo7g110030.1	0	98.74%
	*Bol045651*	BoPUB42	C08	32,648,599–32,650,459	AtPUB36	*AT3G61390.1*	1 × 10^−134^	94.12%	Bo8g095910.1	3.16 × 10^−35^	100.00%
	*Bol037509*	BoPUB43	Scaffold000024	1,052,543–1,056,193	AtPUB36	*AT3G61390.1*	4 × 10^−92^	91.09%	Bo4g101400.1	0	99.88%
	*Bol003429*	BoPUB44	Scaffold000351	326,763–329,071	AtPUB36	*AT3G61390.2*	4.9 × 10^−324^	81.41%	Bo6g077670.1	0	99.87%
	*Bol009241*	BoPUB45	C03	42,826,034–42,829,000	AtPUB50	*AT5G65500.2*	0	85.15%	Bo3g099120.1	0	98.79%
	*Bol019041*	BoPUB46	Scaffold000133	89,160–92,092	AtPUB50	*AT5G65500.1*	0	85.44%	Bo9g018750.1	4.65 × 10^−26^	99.41%
	*Bol017004*	BoPUB47	C06	37,382,487–37,385,659	AtPUB51	*AT5G61560.1*	0	87.61%	Bo7g098800.1	0	99.75%
	*Bol007790*	BoPUB48	Scaffold000256	377,456–380,990	AtPUB51	*AT5G61560.1*	2 × 10^−144^	88.98%	Bo2g161490.1	0	99.35%
	*Bol029355*	BoPUB49	C09	17,336,879–17,341,604	AtPUB53	*AT5G51270.2*	1 × 10^−93^	80.92%	Bo2g050530.1	6.05 × 10^−103^	83.76%
	*Bol045287*	BoPUB50	Scaffold000001_P2	1,247,247–1,250,408	AtPUB53	*AT5G51270.1*	5 × 10^−137^	80.33%	Bo2g050530.2	0	100.00%
	*Bol029573*	BoPUB51	C03	13,545,754–13,549,222	AtPUB54	*AT1G01680.1*	3 × 10^−38^	81.06%	Bo3g039080.1	0	99.48%
	*Bol040674*	BoPUB52	C05	39,733–41,926	AtPUB54	*AT1G01680.1*	0	82.33%	Bo5g001070.1	5.90 × 10^−116^	98.72%
	*Bol040676*	BoPUB53	C05	45,765–48,401	AtPUB55	*AT1G01660.1*	1 × 10^−131^	89.67%	Bo5g001050.1	1.02 × 10^−93^	100.00%
	*Bol013021*	BoPUB54	C03	54,964,336–54,966,942	AtPUB56	*AT1G01670.1*	2 × 10^−99^	82.13%	Bo3g175980.2	0	99.06%
	*Bol040675*	BoPUB55	C05	43,012–45,429	AtPUB56	*AT1G01670.1*	0	79.74%	Bo5g001060.1	0	98.39%
	*Bol000206*	BoPUB56	Scaffold001134	1549–4155	AtPUB56	*AT1G01670.1*	2 × 10^−99^	76.52%	Bo3g175980.1	0	99.06%
	*Bol013313*	BoPUB57	C07	19,000,520–19,002,951	AtPUB57	*AT1G56030.1*	9 × 10^−146^	77.54%	Bo6g052570.1	0	99.54%
	*Bol037990*	BoPUB58	C05	4,401,778–4,406,478	-	*AT5G57035.1*	4 × 10^−167^	89.16%	Bo9g130630.1	0	99.79%
**III**	*Bol021972*	BoPUB59	C01	18,150,442–18,151,785	AtPUB3	*AT3G49810.1*	0	87.31%	Bo1g081270.1	0	100.00%
	*Bol009126*	BoPUB60	C04	8,207,638–8,208,912	AtPUB22	*AT3G52450.1*	0	89.63%	Bo4g125390.1	0	100.00%
	*Bol005035*	BoPUB61	C06	38,049,040–38,050,311	AtPUB22	*AT3G52450.1*	0	87.78%	Bo7g101510.1	0	100.00%
	*Bol025043*	BoPUB62	C08	27,739,954–27,741,261	AtPUB22	*AT3G52450.1*	0	89.14%	Bo8g080650.1	0	99.92%
	*Bol039769*	BoPUB63	C03	8,905,416–8,906,642	AtPUB23	*AT2G35930.1*	0	88.13%	Bo3g029820.1	0	99.02%
	*Bol011695*	BoPUB64	C04	3,917,811–3,919,031	AtPUB23	*AT2G35930.1*	0	88.69%	Bo4g036170.1	0	99.10%
	*Bol037760*	BoPUB65	C04	36,502,645–36,503,883	AtPUB23	*AT2G35930.1*	0	89.55%	Bo4g185810.1	0	99.76%
	*Bol007270*	BoPUB66	C01	36,294,678–36,296,310	AtPUB24	*AT3G11840.1*	0	82.21%	Bo1g139780.1	0	97.57%
	*Bol010350*	BoPUB67	C05	30,543,476–30,545,255	AtPUB24	*AT3G11840.1*	0	83.26%	Bo5g135090.1	0	98.08%
	*Bol003032*	BoPUB68	Scaffold000366	135,192–137,357	AtPUB24	*AT3G11840.1*	0	78.76%	Bo3g064620.1	0	98.21%
	*Bol031005*	BoPUB69	C01	30,733,021–30,734,274	AtPUB25	*AT3G19380.1*	0	82.42%	Bo1g114760.1	0	100.00%
	*Bol018091*	BoPUB70	C05	23,579,947–23,581,212	AtPUB25	*AT3G19380.1*	0	82.11%	Bo5g115230.1	0	98.18%
	*Bol023451*	BoPUB71	C08	927,641–928,912	AtPUB26	*AT1G49780.1*	0	85.99%	Bo8g011300.1	0	99.61%
	*Bol015885*	BoPUB72	C03	30,604,113–30,605,372	AtPUB27	*AT5G64660.1*	0	86.47%	Bo3g101050.1	0	99.68%
	*Bol019081*	-	Scaffold000133	386,596–386,904	AtPUB27	*AT5G64660.1*	6 × 10^−25^	81.32%	Bo9g018240.1	1.44 × 10^−122^	99.58%
	*Bol030961*	BoPUB73	C01	31,316,399–31,317,652	AtPUB29	*AT3G18710.1*	7 × 10^−144^	79,67%	Bo1g116350.1	0	100.00%
	*Bol022877*	BoPUB74	C03	23,114,355–23,115,629	AtPUB29	*AT3G18710.1*	6 × 10^−151^	82.50%	Bo3g071710.1	0	99.29%
	*Bol002996*	BoPUB75	Scaffold000367	136,856–138,127	AtPUB29	*AT3G18710.1*	3 × 10^−121^	81.29%	Bo5g117170.1	0	99.29%
	*Bol005722*	BoPUB76	C08	82,316–83,647	AtPUB31	*AT5G65920.1*	0	80.37%	Bo9g018990.1	0	99.92%
**IV**	*Bol028991*	BoPUB77	C01	1,265,612–1,268,155	AtPUB5	*AT4G36550.1*	0	84.71%	Bo1g005580.1	6.09 × 10^−15^	100.00%
	*Bol018658*	-	C06	47,377,417–47,379,131	AtPUB5	*AT4G36550.1*	8 × 10^−75^	82.40%	Bo7g118020.1	0	98.65%
	*Bol016339*	BoPUB78	Scaffold000156_P2	219,216–221,685	AtPUB5	*AT4G36550.1*	0	82.34%	Bo3g154110.1	0	98.77%
	*Bol016347*	BoPUB79	Scaffold000156_P2	281,312–283,639	AtPUB5	*AT4G36550.1*	0	78.78%	Bo3g154230.1	0	99.86%
	*Bol005289*	BoPUB80	C03	43,163,200–43,166,241	AtPUB6	*AT1G24330.1*	0	85.35%	Bo3g141280.1	0	100.00%
	*Bol041756*	BoPUB81	C06	16,043,256–16,046,166	AtPUB6	*AT1G24330.1*	0	85.86%	Bo7g050100.1	7.04 × 10^−54^	99.77%
	*Bol010460*	BoPUB82	C02	11,448,388–11,451,770	AtPUB7	*AT1G67530.1*	0	87.04%	Bo2g055940.1	0	99.32%
	*Bol008441*	BoPUB83	C03	43,797,474–43,800,768	AtPUB45	*AT1G27910.1*	0	86.75%	Bo3g142960.1	0	99.48%
	*Bol002093*	BoPUB84	Scaffold000404	92,644–95,688	AtPUB45	*AT1G27910.1*	0	89.30%	Bo5g052370.1	0	100.00%
**V**	*Bol023972*	BoPUB85	C07	7,790,036–7,793,449	AtPUB42	*AT1G68940.1*	0	88.81%	Bo6g108490.1	6.60 × 10^−26^	99.47%
	*Bol027639*	BoPUB86	C07	1,710,724–1,713,541	AtPUB43	*AT1G76390.1*	0	83.79%	Bo8g070380.1	0	98.13%
	*Bol007008*	BoPUB87	C08	21,666,213–21,669,713	AtPUB44	*AT1G20780.1*	0	76.65%	Bo8g070380.2	0	98.13%
	*Bol025462*	BoPUB88	Scaffold000087_P1	203,738–207,119	AtPUB44	*AT1G20780.1*	0	84.14%	Bo7g061370.1	0	99.09%
	*Bol011584*	-	C08	2,691,591–2,692,573	-	*AT1G04555.1*	4 × 10^−43^	83.00%	Bo8g003030.1	0	99.33%
**VI**	*Bol007668*	BoPUB89	C05	32,161,132–32,163,836	AtCHIP	*AT3G07370.1*	1 × 10^−140^	88.48%	Bo5g141580.1	0	99.30%
	*Bol033995*	BoPUB90	Scaffold000040	696,720–698,064	AtCHIP	*AT3G07370.1*	0	86.73%	Bo3g061190.1	7.27 × 10^−124^	100.00%
	*Bol040899*	BoPUB91	C05	1,223,432–1,227,503	MAC3A/59	*AT1G04510.1*	0	91.68%	Bo5g004640.1	0	99.81%
	*Bol027262*	BoPUB92	C04	20,160,334–20,165,996	MAC3B/60	*AT2G33340.1*	0	90.63%	Bo4g043800.1	0	99.51%
	*Bol037940*	BoPUB93	C04	35,183,234–35,187,431	MAC3B/60	*AT2G33340.1*	0	91.66%	Bo4g181820.1	0	100.00%
**VII**	*Bol005348*	BoPUB94	Scaffold000301	209,407–210,723	AtPUB21	*AT5G37490.1*	0	83.91%	Bo4g143870.1	0	98.98%
	*Bol004317*	BoPUB95	Scaffold000326	51,300–53,990	AtPUB49	*AT5G67530.1*	0	90.74%	Bo3g096470.1	0	99.05%
	*Bol044051*	BoPUB96	C09	39,181,374–39,183,068	AtPUB62	*AT5G05230.1*	0	85.04%	Bo9g177290.1	0	98.44%
	*Bol034488*	BoPUB97	C03	3,645,792–3,651,503	ATAPY2	*AT5G18280.1*	0	92.86%	Bo3g013060.1	4.81 × 10^−37^	99.56%
	*Bol045810*	-	C08	34,872,922–34,874,905	ATNRAMP3	*AT2G23150.1*	0	83.64%	Bo8g100600.1	0	100.00%
	*Bol021231*	BoPUB98	C02	3,528,313–3,533,241	MUSE3/1	*AT5G15400.1*	0	88.72%	Bo2g012540.1	0	99.17%
	*Bol030454*	BoPUB99	C09	33,424,858–33,429,958	MUSE3/1	*AT5G15400.1*	0	88.06%	Bo9g164140.1	0	99.17%

**Table 2:** Conserved motif of *B. oleracea* U-box gene family determined by MEME software.

Motif	E-Value	Sites	Width	Best Possible Match
1	4.6 × 10^−1084^	82	24	ELMKDPVIVASGQTYERASIZKWL
2	3.5 × 10^−631^	83	21	LSHTTLTPNHTLRSLIQEWCE
3	2.0 × 10^−463^	34	40	IPPLVDLLENGTPRGKKDAATALFNLSIYQENKGRIVRAG
4	2.4 × 10^−413^	91	11	IPEEFRCPISL
5	1.9 × 10^−367^	54	29	MEARENAAATLFSLSVLEENKHKIGSSGA
6	1.3 × 10^−353^	59	29	GVVPSLVKJSQNGTERARRKAASLLKLLR
7	3.2 × 10^−248^	64	11	GHNTCPKTKQV
8	1.1 × 10^−223^	58	21	LLRSGSPRGKENAVAVLLQLC
9	3.3 × 10^−365^	17	50	LAKRNTDNRIVIAEAGAIPLLVNLLKSEDSATQENAVTALLNLSIYENNK
10	1.1 × 10^−178^	12	32	DLKSRKAIYVSREAPDCCHIWFTCKGYLIHTR
11	6.9 × 10^−203^	28	31	BSVEEAILELIYESGIKKLVMGAAADRHYSR
12	4.2 × 10^−195^	11	50	LQEMLQLGVVAKLCLVLQVSCGNKTKEKAKELLKLHARVWKESPCIPRNL
13	2.1 × 10^−182^	14	41	DEKIYVAVNKDVEESKSTLVWALRNLGGKKJCJLHVHQPIS
14	2.4 × 10^−176^	9	50	QCAEGRAEFLNHGAAIAVVTKKILRVSQIASDRAVRVLLSVGRFCATPAL
15	1.4 × 10^−168^	49	21	VEEALAVLANLASHPEGKEAI

**Table 3:** Analysis of the KEGG pathway of U-box gene in *B. oleracea*.

NCBI-Gene ID:	Pathway ID	Description	Gene Name	Bo U-Box Transcriptome ID
hsa:27339	hsa04120	Ubiquitin mediated proteolysis	*PRPF19, NMP200, PRP19, PSO4, SNEV, UBOX4, hPSO4*	Bo5g004640.1 Bo4g181820.1 Bo4g043800.1
	hsa03040	Spliceosome		
hsa:10277	hsa04120	Ubiquitin mediated proteolysis	*UBE4B, E4, HDNB1, UBOX3, UFD2, UFD2A*	Bo9g164140.1 Bo2g012540.1
	hsa04141	Protein processing in endoplasmic reticulum		
hsa:10273	hsa04120	Ubiquitin mediated proteolysis	*STUB1, CHIP, HSPABP2, SCAR16, SDCCAG7, UBOX1*	Bo5g141580.1 Bo5g135090.1 Bo3g061190.1
	hsa04141	Protein processing in endoplasmic reticulum		
hsa:23759	hsa04120	Ubiquitin mediated proteolysis	*PPIL2, CYC4, CYP60, Cyp-60, UBOX7, hCyP-60*	Bo3g096470.1

## Supplementary Materials

The following are available online at https://www.mdpi.com/2073-4425/10/12/1000/s1. Table S1: Primers for RT-qPCR analysis of selected U-box genes in *B. oleracea*, Table S2: Tandem replication gene pairs of the U-box family members in the *B. oleracea* genome and between the *B. oleracea* and *A. thaliana* genomes, Table S3-1: Tandem repeat gene Ka/Ks value in the *B. oleracea* genome, Table S3-2: Tandem repeat gene Ka/Ks value between the *B. oleracea* and *A. thaliana* U-box genomes, Table S4: GO Annotation Analysis of U-box genes in *B. oleracea*, Table S5: Expression levels of the U-box gene in *B. oleracea* after different pollination conditions and time, Table S6: Cis-Acting elements analysis in the 1.5 kb promoters of U-box genes in *B. oleracea*.
